# Comparison of effects of 0.5 % levobupivacaine with buprenorphine and nalbuphine in ultrasound-guided supraclavicular brachial plexus block- A Randomised Control Trial

**DOI:** 10.12688/f1000research.164457.1

**Published:** 2025-07-03

**Authors:** Suvajit Podder, Deepali Shetty, Shweta Sinha, Shwetha Krishna, Souvik Chaudhuri, Mahesh Nayak, Aditya Rameshbabu Devalla, Jesica Maria

**Affiliations:** 1 Department of Anaesthesiology, Kasturba Medical College, Manipal, Manipal Academy of Higher Education, Manipal, Karnataka, 576104, India; 2 Department of Critical Care Medicine, Kasturba Medical College, Manipal, Manipal Academy of Higher Education, Manipal, Karnataka, 576104, India

**Keywords:** Levobupivacaine, Nalbuphine, Buprenorphine, Brachial Plexus Block, Orthopedic Procedures

## Abstract

**Introduction:**

The brachial plexus block is one of the most commonly used anaesthesia techniques for upper limb surgeries. The supraclavicular brachial plexus block (SCBB) has been a frequently performed technique because it provides excellent quality of blockade, faster onset, and dense blockade. Different adjuvants and local anaesthetics are used to improve the onset and quality of the blockade. In our study, the local anaesthetic used was 0.5% levobupivacaine, and the adjuvants used were buprenorphine and nalbuphine.

**Materials and Methods:**

Our study, a randomised, prospective double-blinded investigation, was conducted on 60 patients scheduled for elective upper limb orthopaedic surgeries. We performed the Supraclavicular brachial plexus block with 25 ml of 0.5% levobupivacaine plain (Group L) or with buprenorphine (Group LB) or with nalbuphine (Group LN) as adjuvants. We recorded the onset, duration of blockade (motor and sensory), sedation score, and time for rescue analgesia.

**Results:**

The onset of sensorimotor blockade was notably faster in Group LB than in Group LN or Group L. The sensorimotor blockade duration was significantly prolonged in Group LB compared to LN and L. Group LB also had a delayed time after 12 hrs for rescue analgesia compared to the other two groups.

**Conclusion:**

Buprenorphine as an adjuvant to 0.5% levobupivacaine in ultrasound-guided supraclavicular brachial plexus block significantly shortens the onset of sensorimotor blockade and enhances the duration of blockade when compared to nalbuphine. Both the adjuvants delayed the time for request of rescue analgesia compared to plain levobupivacaine.

## Introduction

Regional anesthesia has gained popularity and has been extensively employed in current clinical practice. Its safety, cost-effectiveness, and ability to maintain hemodynamic stability while providing prolonged postoperative pain relief make it a cornerstone of anesthesia. In 1885, William Stewart Halsted performed the first brachial plexus block under direct vision at Roosevelt Hospital in New York City. ^1^ This technique, particularly brachial plexus block, is the preferred choice for upper limb surgeries because of its safety and effectiveness.

Supraclavicular brachial plexus block (SCBB) has emerged as a popular technique, largely due to its superior blockade quality, rapid onset, and dense blockade. ^2^ The introduction of ultrasound guidance has significantly enhanced the accuracy and safety of the procedure, aiding in the visualization of normal and abnormal anatomical variations and the spread of local anesthetics. This technological advancement has notably improved the quality and success rate of the block, providing anesthesiologists with a powerful tool for patient care. ^3^

Different adjuvants, such as opioids, clonidine, dexmedetomidine, ketamine, midazolam, neostigmine, and dexamethasone, are used along with local anesthetics to improve the onset and quality of the blockade. In our unique study, we aimed to compare the block characteristics of the adjuvants buprenorphine and nalbuphine, along with the local anesthetic agent 0.5% levobupivacaine for supraclavicular brachial plexus block, which had never been compared together. We noted the onset and duration of sensorimotor blockade as the primary objective. The secondary objectives were to determine the time required for rescue analgesia, sedation scores, and adverse effects.

## Methods and materials

This randomized, prospective, double-blinded trial was conducted in a tertiary hospital in accordance with the Declaration of Helsinki (2013). We started our study after obtaining approval from the Departmental scientific committee and Kasturba Medical College and Kasturba Hospital institutional ethics committees (IEC: 453/2020; date of approval-12/08/2020). This study was registered with the Clinical Trials Registry-India (CTRI) -CTRI/2021/06/034424, date of approval: 28/06/2021 ( https://ctri.nic.in). Participants were explained in their own understandable language about our trial, and informed written consent was taken from all participants included in the trial after satisfying the inclusion and exclusion criteria.

Sixty patients who were scheduled for elective upper limb orthopaedic surgeries under ultrasound-guided supraclavicular brachial plexus block belonging to the American Society of Anesthesiologists physical status (ASA PS) I or II, aged 18–60 years of either sex, were included, in the study. Participants who refused to participate, had a history of allergy to the study drugs, seizure disorder, or any pre-existing neurological deficits, and pregnant women were excluded from our study.

Depending on the group allocated, the anesthesiologist consultant posted in the OT administered the study drug using an ultrasound machine.

The study included three observers:

Observer 1- A Postgraduate randomized the patients into three groups using a computer-generated table. The same anesthesiologist was no longer involved in this study. The groups were allocated as follows: **Group L** received 24 ml of 0.5% levobupivacaine +1 ml saline. **Group LB** received 24 ml of 0.5% levobupivacaine with 300 μg (1 ml) buprenorphine. **Group LN** received 24 ml of 0.5% levobupivacaine with 10 mg (1 ml) of nalbuphine. The block was given by consultant experienced in USG guided brachial plexus block who was the Observer 2. The 3 ^rd^ observer- Nursing staff in the postoperative unit who assessed the block and was blinded to the procedure and the drug given.

**Figure 1.**
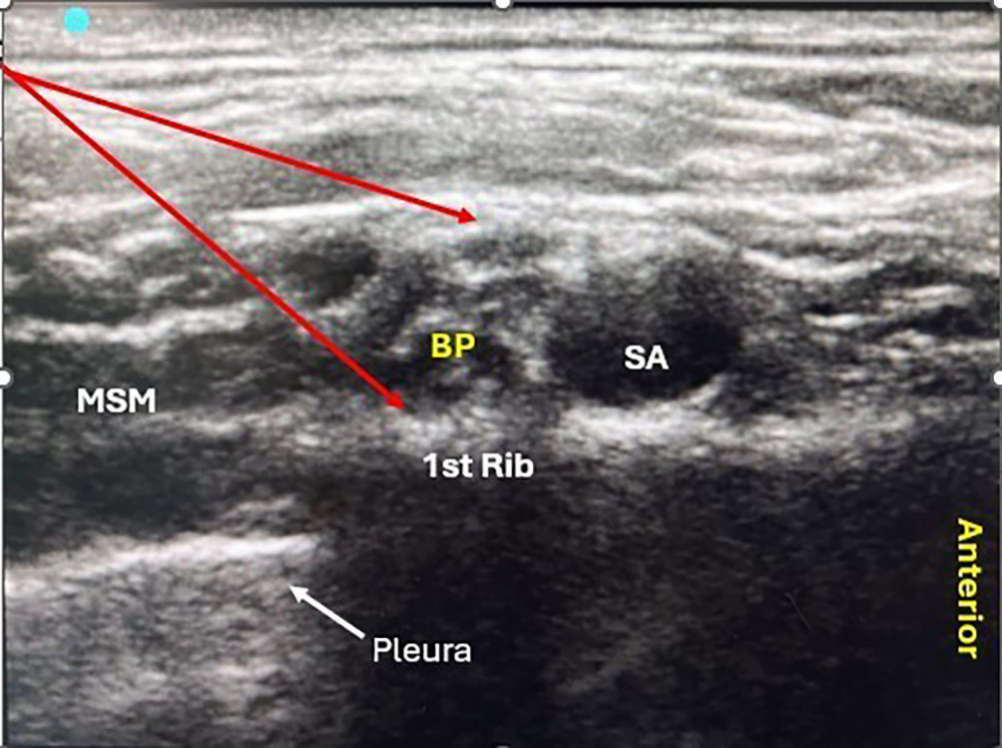
Sono-Anatomy of the supraclavicular block (BP- Brachial Plexus, SA- Subclavian Artery, MSM- Middle Scaleni Muscle).

Pre-anesthetic evaluation was performed the day before surgery. On the day of surgery, the NPO status was confirmed. The patients baseline heart rate, blood pressure, and oxygen saturation were recorded. An intravenous cannula was then secured to the unaffected limb. The block was performed in a supine position with a 45 ^°^ head-up position, and the patient’s head was turned towards the unaffected side. Under sterile aseptic precautions, the consultant anesthesiologist administered the ultrasound-guided supraclavicular block via an in-plane technique (IP) ( Figure 1). After administration of the block, the onset and duration of sensorimotor blockade and sedation score were recorded.

The onset of sensory block was noted from the time interval between the administration of local anesthetic solutions to the loss of prick sensation using a toothpick on C5,6,7 dermatomes. It was performed every 3 min for the next 30 min until the onset of the loss of sensation. Sensory block was graded on a 3-point scale: Grade 0, sharp prick felt; Grade 1, analgesia, dull sensation felt; Grade 2, anesthesia; and no sensation felt. ^4^ The onset of motor blockade was defined as the interval between the administration of the local anesthetic solution and the loss of movements of the ipsilateral upper limb. The onset of motor blockade was assessed every 3 min for 30 min using a modified Bromage scale for the upper extremities. Modified Bromage scale (3-point scale): Grade 0, normal motor function; Grade 1, decreased motor function with the ability to move only fingers; Grade 2, complete motor block with the inability to move fingers. ^5^

The duration of sensory block was noted from the loss of sensation to the toothpick prick to the reappearance of the prick sensation. The duration of the motor block was defined as the interval between complete motor blockade and the appearance of finger movements. The time of rescue analgesia was noted from the administration of medication (block) to the request for any pain-relieving medications by the patient. Sedation scores were assessed postprocedure using the sedation score described by Culebras et al. 1-awake and alert, 2-sedated, responding to verbal stimulus, 3-sedated, responding to mild physical stimulus, 4-sedated, responding to moderate to severe physical stimulus, 5- Not arousable. ^6^ Any adverse events that occurred after blocking were meticulously documented.

After 30 min of administering the block with the study drug, if the sensory blockade was less than grade 2 (anesthesia, no sensation felt), it was considered an unsuccessful block. The same participants were administered general anesthesia according to the department protocol, and these patients were excluded from the study. Postoperatively, rescue analgesia as 1 mg/kg tramadol for a VAS score between 4 and 6. For severe pain (VAS score > 6), intravenous nalbuphine (1 mg/kg) was administered.

### Sample size

This study enrolled a total of sixty patients, with 20 in each group.

**Analysis:** A priori: Compute required sample size

**Input:** Effect size f = 0.42, α error = 0.05, power (1-β error) = 0.80, number of groups = 3

**Output:** Non-centrality parameter λ = 10.5840000, Critical F = 3.1588427, degree of freedom:numerator df = 2, denominator df = 57, total sample size = 60, actual power = 0.8169259

The sample size was estimated using G*Power software v. 3.1.9.4 (Franz Faul, Universität Kiel, Germany). Considering the effect size to be measured (f ) at 42%, the power of the study at 80%, and the alpha error at 5%, the sample size needed was 60, with each study group comprising 20 samples.

The patients were randomised into one of the two parallel groups and they will be allocated 1:1 using a computer-generated software program ( https://www.sealedenvelope.com) with block randomization of varying size blocks ^2, 4, 8^

For the analysis of the data gathered from the study, the Statistical Package for Social Sciences (SPSS) 22.0 data package was used. The data were analyzed using the chi-square test, Kruskal-Wallis test, and one-way ANOVA. The level of significance ( *p* < 0.05) was used in all tests in the study.

## Results

60 participants were enrolled in our trial, with 20 participants each in Group L, LB and LN.

The Demographic variables were comparable between groups L, LB, and LN ( Table 1).

**Table 1.** Demographic variables.

Variables	Group L (n = 20)	Group LB (n = 20)	Group LN (n = 20)	p-Value
**Age (Years) (Mean ± SD)**	41.00 ± 7.81	39.80 ± 9.42	40.55 ± 11.01	0.90 ^#^
**BMI (kg/m** ^**2**^ **) (Mean ± SD)**	23.08 ± 2.72	21.43 ± 3.04	21.48 ± 3.55	0.17 ^#^
**Gender (Male/Female)**	9/11	10/10	14/6	0.24 ^*^
**ASA-PS (1/2)**	15/5	17/3	14/6	0.52 ^*^

^#^ One-way ANOVA.

* Chi-square Test.

The onset of the sensory block was faster in Group LB (6.80 min) than in Group LN (9.60 min) and Group L (11.60 min) and was statistically significant ( *p*-Value of < 0.001). Similarly, a comparison of the onset of the motor block also showed a faster onset in Group LB (9.30 minutes compared to Groups LN and B which required 15.45 minutes and 19.65 minutes, respectively.

The duration of the sensory blockade was longer in Group LB with 12.2 hrs compared to Group LN and Group L, which were 9.8 hrs and 7.3 hrs, respectively, and it was found to be statistically significant. Similarly, the duration of the motor block was also longer in Group LB with 8.4 hrs, compared to Group LN and Group L which was 7.8 hrs and 5.6 hrs and was also statistically significant.

The time to ask for rescue analgesia in the postoperative period was later in Group LB (13.9 hrs) than in Groups LN and L (13.4 hrs and 8.5 hrs, respectively). The sedation scores were similar in all three groups ( Table 2).

**Table 2.** Onset of the sensory-motor block, sedation score and duration of surgery.

		Group L (n = 20)	Group LB (n = 20)	Group LN (n = 20)	*p*-Value
		Mean ± SD	Mean ± SD	Mean ± SD	
**Sensory onset (min)**		11.60 ± 3.09	6.80 ± 1.54	9.60 ± 1.23	<0.001 ^#^
**Motor onset (min)**		19.65 ± 2.28	9.30 ± 2.36	15.45 ± 1.10	<0.001 ^#^
**Sedation score (Median Score)**	**Median**	1	1	1	1 ^##^
	**IQR**	(1-1)	(1-1)	(1-1)	
**Duration of surgery (min) (Mean ± SD)**		79.25 ± 35.22	84.50 ± 53.60	78.00 ± 36.79	0.96 ^#^

^#^ One-way ANOVA.

^##^ Kruskal-Wallis Test.

## Discussion

Supraclavicular brachial plexus block (SCBB) is widely regarded as spinal anesthesia of the upper extremity, as it provides excellent analgesia, anesthesia, and surgical relaxation for upper limb surgeries. Local anesthetic agents bind to voltage-gated Na+ channels and disrupt neuronal transmission. ^7^

Adjuvants, when added to local anesthetic agents, act synergistically. They decrease the onset of blockade, extend the duration of sensory and motor blockade, reduce the requirement for additional sedatives or analgesics, and decrease the cumulative dose of local anesthetic agents. ^8^ Despite the use of long-acting amide local anesthetic drugs, the use of additional adjuvants that can prolong the duration of surgical blockade and continue to provide postoperative analgesia after motor blockade has worn off.

Our study aimed to fill this gap by comparing the unique properties of buprenorphine and nalbuphine as adjuvants to 0.5% levobupivacaine in SCBB. Buprenorphine, a partial mu receptor agonist, weak kappa receptor antagonist, and delta receptor antagonist, acts on the central nervous system and has a potent analgesic effect. ^9^ Nalbuphine, a mu receptor antagonist and kappa receptor agonist, inhibits the release of neurotransmitters, such as substance P, which are involved in pain transmission. ^10^

**Figure 2.**
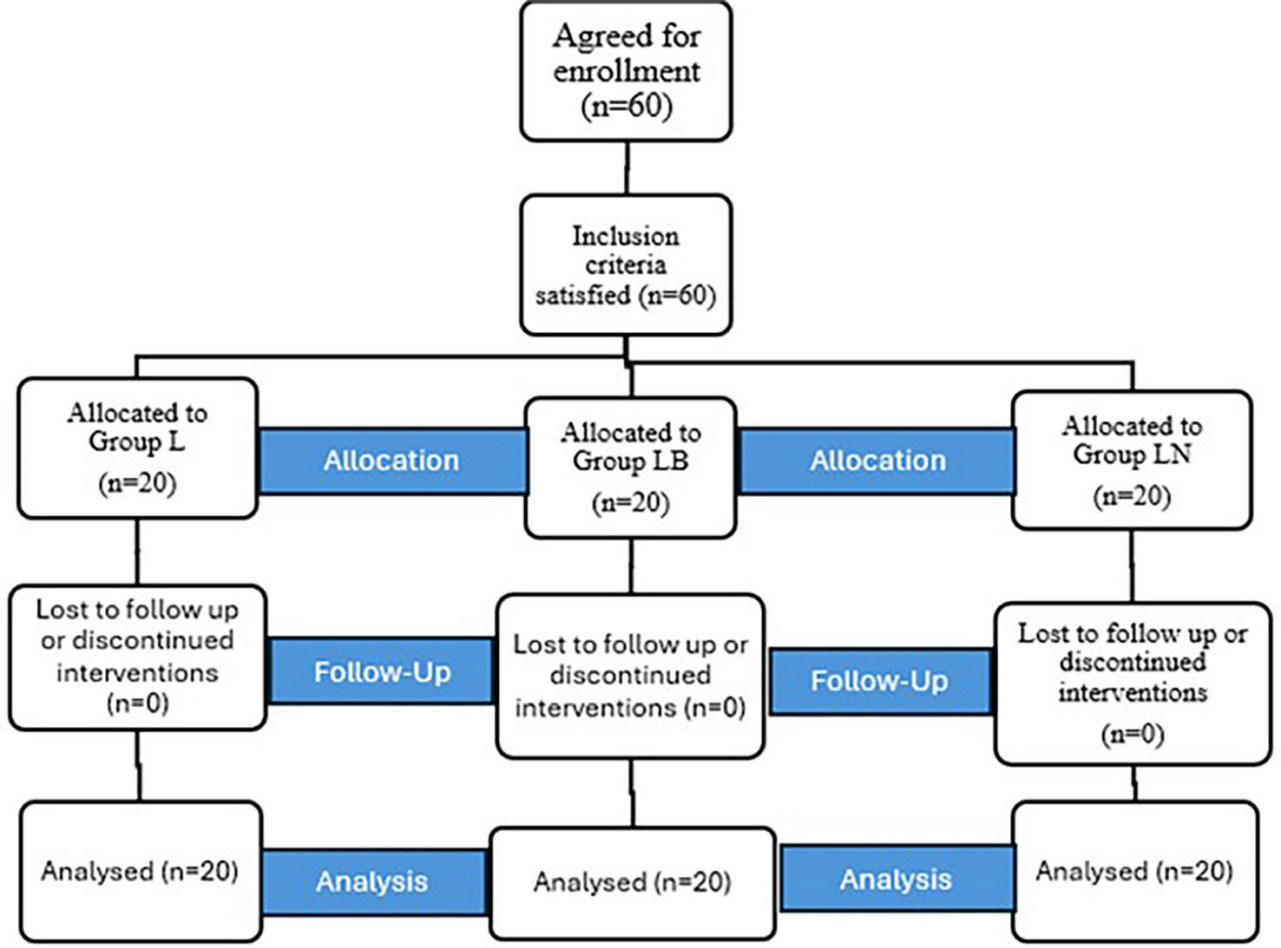
CONSORT flow diagram.

Sixty patients were allocated to our study for the USG-guided supraclavicular block and where randomised as depicted in the CONSORT flow diagram ( Figure 2). There were no failed blocks and no dropouts. Our primary objectives were to determine the onset and duration of sensory and motor blocks after administering SCBB.

We noted a quicker onset of sensory blockade in Group LB (6.80 ± 1.54 min) than in Group LN (9.60 ± 1.23 min) and Group L (11.60 ± 3.09 min) ( Table 2). Similarly, the onset of motor blockade was also quicker in Group LB (9.30 ± 2.36 min) than in Group LN (15.45 ± 1.10 min) and Group L (19.65 ± 2.28 min) ( Table 2). Similarly, Qureshi et al. reported that nalbuphine as an adjuvant with a local anesthetic agent had a faster onset of sensorimotor action, comparable to our trial. ^11^ A study by Patil et al. found the result to be different from our study, where they found that both the sensory and motor onset were the same regardless of whether buprenorphine was used as an adjuvant. ^12^ Sanghvi et al. found a contrasting result when comparing the time of onset of sensory-motor block with buprenorphine as an adjuvant, and they did not note any statistical difference.

**Figure 3.**
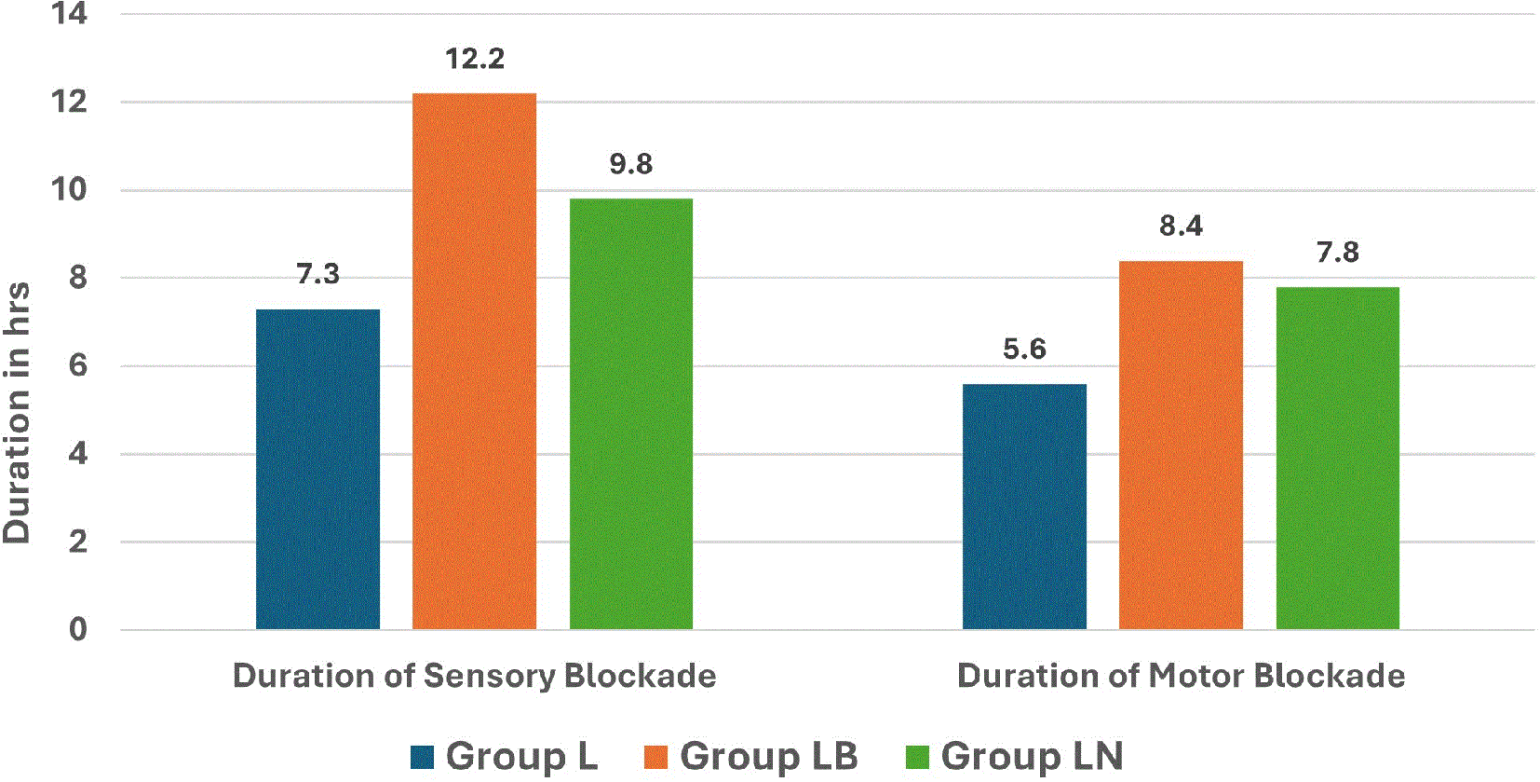
Duration of sensory and motor blockade.

Similarly, the duration of the sensory blockade was significantly longer in Group LB (12.2 hrs) than in both Group LN (9.8 hrs) and Group L (7.3 hrs) ( Figure 3). We also noted that the duration of motor blockade was significantly prolonged in Group LB (8.4 hrs) compared to Group LN (7.8 hrs) and Group L (5.6 hrs) ( Figure 3). Similarly, Qureshi et al. reported that the duration of block with nalbuphine was similar to that in our trial; the sensory block was for 12 h, and the motor block was for 11 h. ^11^ However, Patil et al. did not find any significant difference in sensory-motor duration if buprenorphine was used as an adjuvant with a local anesthetic agent. ^12^

**Figure 4.**
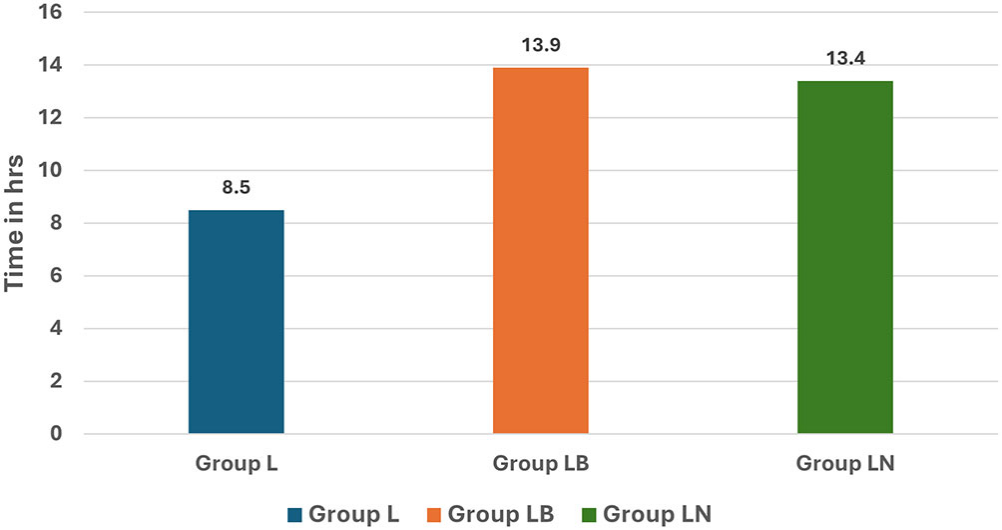
Time to rescue analgesia.

The time of rescue analgesic request by the patients was once again significantly longer in Group LB (13.9 h) than in groups LN (13.4 h) and L (8.5 h) ( Figure 4). Our results obtained by us were consistent with those obtained by Patil et al. ^12^ They also concluded that buprenorphine, when added to bupivacaine, prolonged the duration of postoperative analgesia and time to rescue analgesia when compared to the control group without any adjuvant. Similar outcomes were obtained by Jadon et al., who concluded that the addition of buprenorphine (3 mcg/kg) to 0.3% bupivacaine effectively increased the duration of sensory block and analgesia and improved the quality of the block without affecting the motor component. ^13^ Candido et al. also reported that the addition of buprenorphine (17.4 ± 1.26 h) to local anesthetics in patients undergoing upper limb surgery under subclavian perivascular brachial plexus block provides a 3-fold longer duration of analgesia when compared to the local anesthetic alone. ^14^ Similarly, Abdelhaq and Elramely et al. performed a study with 20 mg nalbuphine with local anesthetic agents and found that the time for rescue analgesia was similar to that in our study after 13 h, whereas we achieved the same time of analgesia with a low dose of 10 mg of nalbuphine. ^15^

Thus, our study further confirms that buprenorphine significantly prolongs the duration of postoperative analgesia, decreases the need for systemic analgesics, and prevents unwanted side effects. In addition, our study also found nalbuphine to be an acceptable agent for use as an adjuvant in supraclavicular brachial plexus blocks, as it also prolonged the duration of sensorimotor blockade significantly compared to the local anesthetic alone. Although the addition of buprenorphine provided much longer sensory and motor blocks than the addition of nalbuphine, the time for rescue analgesic request was not much longer in the buprenorphine group than in the nalbuphine group. This suggests that nalbuphine offers a post-operative analgesic profile comparable to buprenorphine, but with a shorter duration of motor effects. Therefore, it may be a suitable adjunct for shorter-duration upper limb surgeries, in which early recovery of motor function is crucial. This finding opens new possibilities for pain management in shorter surgeries, offering a more optimistic and forward-thinking approach. Further studies should aim to identify the effect of adding different doses of nalbuphine while titrating it according to body weight to local anesthetics for supraclavicular brachial plexus blocks and evaluate its effect on nerve block duration and rescue analgesic request time.

## Conclusion

Buprenorphine as an adjuvant to 0.5% levobupivacaine in supraclavicular brachial plexus block significantly shortens the onset of sensorimotor blockade and enhances the duration of blockade compared to nalbuphine. Both adjuvants delayed the time required for rescue analgesia compared to plain levobupivacaine.

Limitation—In our study, we observed that opioids as adjuvants helped improve block characteristics, but the study was performed on a very limited sample. Therefore, similar studies should be performed with a larger sample size to better understand the role of adjuvants in regional anesthesia.

### Ethics and consent statement

The research followed the tenets of the Declaration of Helsinki. The institutional ethical committee namely Kasturba Medical College and Kasturba Hospital Institutional Ethics Committee (IEC 453/2020) on 12 ^th^ August 2020.

Participants were explained in their own understandable language about our trial, and informed written consent was taken from all participants included in the trial after satisfying the inclusion and exclusion criteria.

## Data Availability

Figshare: This study contains the underlying data for “Comparison of effects of 0.5 % levobupivacaine with buprenorphine and nalbuphine in ultrasound-guided supraclavicular brachial plexus block- A randomised control trial”. ( https://figshare.com/articles/dataset/Protocol_doc/28910759) **DOI: (** **https://doi.org/10.6084/m9.figshare.28910759.v2** ) ^16^
• Data OI. xlsx Data are available under the terms of the Creative Commons Attribution 4.0 International license (CC-BY 4.0). Figshare: Extended data for **COMPARISON OF EFFECTS OF 0.5 % LEVOBUPIVACAINE WITH BUPRENORPHINE AND NALBUPHINE IN ULTRASOUND-GUIDED SUPRACLAVICULAR BRACHIAL PLEXUS BLOCK- A RANDOMISED CONTROL TRIAL** ( https://figshare.com/articles/dataset/Protocol_doc/28910759) This project contains the following extended data:
• Consort checklist • Proforma • Protocol **DOI**: **(** **https://doi.org/10.6084/m9.figshare.28910759.v2** ) ^16^ Data are available under the terms of the Creative Commons Attribution 4.0 International license (CC-BY 4.0).
