# Supplementary material for: Comparison of effects of 0.5 % levobupivacaine with buprenorphine and nalbuphine in ultrasound-guided supraclavicular brachial plexus block- A Randomised Control Trial
Source: F1000Res. 2025 Dec 18;14:646. Originally published 2025 Jul 3. [Version 2] doi: 10.12688/f1000research.164457.2 (PMC12775666; doi:10.12688/f1000research.164457.2)
Supplement: Peer review history

# Reviewer report: Reviewer response for version 2

**DOI:** 10.5256/f1000research.192523.r442883

**Published:** 2026-01-07

**Authors:** Siri Kandavar

**Affiliations:** 1 KS Hegde Medical Academy, Karnataka, India

Accepted

Is the work clearly and accurately presented and does it cite the current literature?

Yes

If applicable, is the statistical analysis and its interpretation appropriate?

I cannot comment. A qualified statistician is required.

Are all the source data underlying the results available to ensure full reproducibility?

Yes

Is the study design appropriate and is the work technically sound?

Yes

Are the conclusions drawn adequately supported by the results?

Yes

Are sufficient details of methods and analysis provided to allow replication by others?

Yes

Reviewer Expertise:

Airway management

I confirm that I have read this submission and believe that I have an appropriate level of expertise to confirm that it is of an acceptable scientific standard.

# Reviewer report: Reviewer response for version 1

**DOI:** 10.5256/f1000research.180977.r399126

**Published:** 2025-11-25

**Authors:** Siri Kandavar

**Affiliations:** 1 KS Hegde Medical Academy, Karnataka, India

1. sample size calculation is mentioned but it is not clear which primary outcome was used to derive the effect size .2. Randomisation information is unclear 

 3. Randomisation and blinding clarification needed . 

 4. Provide complete statistical data (mean ± SD, p-values, CI). 

 5. Figure 3 and 4 are not shown  in the article 

 6. Details on whether block success was confirmed by ultrasound spread or not is not mentioned anywhere .

 7.More recent ultrasound-based studies could be included

 8.Standardise terminology (e.g., “hrs” vs “hours”).

Is the work clearly and accurately presented and does it cite the current literature?

Yes

If applicable, is the statistical analysis and its interpretation appropriate?

I cannot comment. A qualified statistician is required.

Are all the source data underlying the results available to ensure full reproducibility?

Yes

Is the study design appropriate and is the work technically sound?

Yes

Are the conclusions drawn adequately supported by the results?

Yes

Are sufficient details of methods and analysis provided to allow replication by others?

Yes

Reviewer Expertise:

Airway management

I confirm that I have read this submission and believe that I have an appropriate level of expertise to confirm that it is of an acceptable scientific standard, however I have significant reservations, as outlined above.

# Response

**Published:** 2025-11-28

**Authors:** Dr Suvajit Podder

**Affiliations:** Anaesthesiology, Kasturba Hospital Manipal, Manipal, Karnataka, India

1. sample size calculation is mentioned but it is not clear which primary outcome was used to derive the effect size - mentioned in the manuscript

 2. Randomisation information is unclear - mentioned in the manuscript

 3. Randomisation and blinding clarification needed - mentioned in the manuscript 

 4. Provide complete statistical data (mean ± SD, p-values, CI).- mentioned in the manuscript 

 5. Figure 3 and 4 are not shown  in the article - highlighted  

 6. Details on whether block success was confirmed by ultrasound spread or not is not mentioned anywhere -mentioned in the manuscript 

 7.More recent ultrasound-based studies could be included- mentioned in the manuscript 

 8.Standardise terminology (e.g., “hrs” vs “hours”).- standardized to hrs

# Response

**Published:** 2025-12-01

**Authors:** Dr Suvajit Podder

**Affiliations:** Anaesthesiology, Kasturba Hospital Manipal, Manipal, Karnataka, India

I have done the changes that was told to me for my previous manuscript. Do let us know, if any more changes are required

# Reviewer report: Reviewer response for version 1

**DOI:** 10.5256/f1000research.180977.r399128

**Published:** 2025-11-25

**Authors:** Vamshidhar Chamala

**Affiliations:** 1 All India Institute of Medical Sciences, Mangalagiri, Andhra Pradesh, India

The adjuvants of the study are commonly used ones and relevant to the current clinical practise. The study design is aptly sound and appropriate statistical analysis has been applied to the study. The results are mostly comparable to the other similar studies. A larger sample size would more clearly define the role of adjuvants.

 Adequate refernces have been mentioned.

Is the work clearly and accurately presented and does it cite the current literature?

Yes

If applicable, is the statistical analysis and its interpretation appropriate?

Yes

Are all the source data underlying the results available to ensure full reproducibility?

Yes

Is the study design appropriate and is the work technically sound?

Yes

Are the conclusions drawn adequately supported by the results?

Yes

Are sufficient details of methods and analysis provided to allow replication by others?

Yes

Reviewer Expertise:

Regional/Trauma/Obstetric Anesthesia

I confirm that I have read this submission and believe that I have an appropriate level of expertise to confirm that it is of an acceptable scientific standard.

# Response

**Published:** 2025-11-25

**Authors:** Dr Suvajit Podder

**Affiliations:** Anaesthesiology, Kasturba Hospital Manipal, Manipal, Karnataka, India

Thank you so much for reviewing our article.
